# Smallpox and Dracunculiasis: The Scientific Value of Infectious Diseases That Have Been Eradicated or Targeted for Eradication. Is Schistosomiasis Next?

**DOI:** 10.1371/journal.ppat.1005298

**Published:** 2016-01-07

**Authors:** Michael H. Hsieh, Margaret M. Mentink-Kane

**Affiliations:** 1 Research and Development, Biomedical Research Institute, Rockville, Maryland, United States of America; 2 Division of Urology, Children’s National Medical Center, Washington, D.C., United States of America; 3 Department of Urology, The George Washington University, Washington, D.C., United States of America; The Fox Chase Cancer Center, UNITED STATES

Scientists and clinicians studying a particular disease have an ideal goal that, if achieved, would be paradoxical: finding the disease cure and thereby putting themselves out of work. 2015 marks the 35th year since the cure for smallpox eradicated this human scourge. Before a vaccine was developed, infection with smallpox virus occurred in over 10 million people per year around the world, with a death rate greater than 30% [1]. The World Health Organization’s (WHO’s) intensive eradication program against smallpox began in 1967 and was maintained for over ten years. The last known case of smallpox was recorded in Somalia in 1977, and WHO declared the global eradication of smallpox a success in 1980. Vaccinia virus (VACV), like smallpox a member of the Poxviridae family, was used to produce the vaccine that led to smallpox eradication. Today, VACV remains a critical research tool and serves as the laboratory model for poxvirus. VACV is currently used for the production of 2nd- and 3rd-generation smallpox vaccines as well as for the exploration of immuno-oncolytic therapies against melanoma and other cancers [2,3]. VACV and its derivatives, including modified vaccinia virus (MVA) and New York attenuated vaccinia virus (NYVAC), are used as vectors by which to induce immunity to pathogens including HIV, *Plasmodium falciparum*, and *Mycobacterium tuberculosis* [4,5]. VACV also continues to be an important tool for examining fundamental viral mechanisms of immune evasion, including viral immunomodulation and inhibition of cell apoptosis, and has aided investigators in characterizing the innate and adaptive host immune response to viral infection [6]. Therefore, despite the eradication of human disease caused by smallpox virus, basic and applied research using the model vaccinia virus continues to guide our understanding of host immune responses, antigen immunogenicity, viral manipulation of host defenses, and vaccine efficacy.

A second infectious pathogen that is targeted for eradication is *Dracunculus medinensis*, or guinea worm, the nematode parasite that causes drancunculiasis. *D*. *medinensis* is contracted by drinking larvae-contaminated water. The parasite matures to an adult worm in the host gut and emerges painfully months later through the skin of the lower extremities. Through efforts of the World Health Assembly (as part of WHO), the Carter Center, and other organizations, dracunculiasis is nearing worldwide eradication. In the 1980s, 20 endemic countries accounted for 3.5 million cases of dracunculiasis; by 2014, only 126 cases were reported in four countires: Chad, Ethiopia, Mali, and South Sudan [7,8]. Despite being close to eradicating *D*. *medinensis*, we continue to use this parasite to understand endosymbiotic relationships in organisms. *Wolbachia*, a bacterial symbiont found in many filarial nematodes and necessary for worm survival, are absent in *D*. *medinensis* [9]. Understanding why most filarial nematodes require *Wolbachia*, through comparative laboratory-based studies of *Drancunculus*, may inform endosymbiont-targeted efforts to eradicate other filarial parasites. Thus, we contend that there is an important scientific role for maintenance of selected pathogens that have otherwise been eradicated, or nearly eradicated, outside the laboratory setting.

Like dracunculiasis, schistosomiasis is a tropical disease caused by a parasitic worm. This snail-borne disease is deeply tied to modifiable environmental conditions (i.e., bodies of water conducive to snail survival), a feature which has, in large part, led to calls to set an agenda for schistosomiasis elimination [10]. Schistosomes are spread by freshwater snails when the larval form of schistosomes, the cercariae, emerges from infected snails and penetrates the skin of its human host, taking up residence in blood vessels following maturation to adult worms. The adult worms produce hundreds of eggs per day, some exiting the host in the urine and/or feces, contributing to the pernicious cycle of environmental contamination and reinfection in people. However, many of the eggs become trapped in host tissues including the liver, intestine, and bladder. The damage caused by chronic parasite egg deposition is significant and contributes to the high morbidity and mortality observed in schistosomiasis. Schistosome infection presents unique challenges to worldwide eradication goals and has even proven difficult to eliminate from a targeted endemic region. If eradication of schistosomiasis were achieved, however, the benefits of schistosomiasis research would remain. In fact, the study of schistosomes, schistosomiasis pathogenesis, and immunological characterizations of the host response to parasite egg antigen are currently large areas of study that have been driven by use of schistosomes as a model organism to study stem cell biology, acute and chronic inflammation, liver and gut fibrosis, and T helper 2-type responses, among many others. These and other research objectives will remain a focus of study for many scientists and clinicians, independently of a “cure” for schistosomiasis.

For example, it has long been observed that schistosomes persist for years to decades in the blood vessels of humans. This successful parasitism underscores *Schistosoma* species’ aptitude for host immune evasion and longevity. In recognition of this interesting property of *Schistosoma* worms, planaria scientists have begun adapting their versatile toolbox to schistosomes, also a flatworm of the phylum Platyhelminthes. Collins et al. [11] have characterized a proliferating somatic cell (PSC) population in adult worms that may contribute to the ability of schistosomes to repair injury and resist senescence. Studying the stem cell biology of long-lived metazoans may reveal pathways relevant to human aging.

Schistosomes have also proven to be powerful tools by which some of the earliest delineation of the TH1 versus TH2 immunity paradigm was shaped [12], with implications for immunological pathway discovery across many diseases. Infection with pathogens that induce TH1 CD4+ lymphocytes and secrete interferon gamma (IFNg) and interleukin-2 (IL-2) can be compared to induction of the TH2 response following schistosome infection or exposure to parasite eggs or egg antigen resulting in the production of IL-4, IL-5, and IL-13. In addition, the mouse model of *Schistosoma mansoni* infection has helped characterize the role of IL-10 as an anti-inflammatory cytokine required to control liver pathology following egg deposition and, more broadly, has helped define IL-10 immunoregulation in other diseases in which inflammation drives pathology, including ulcerative colitis, Crohn’s disease, and, in the lung, allergy and asthma. In addition to schistosomiasis, IL-10 helps control the immunopathogenesis observed following infection with *Plasmodium* spp., *Mycobacterium* spp., and *Trypanosoma cruzi*.

Several investigators have taken advantage of the strong immunogenic properties and ease of use of *S*. *mansoni* parasite eggs and soluble egg antigens (SEA). Graham et al. [13] have modeled pulmonary hypertension following intravenous injection of *Schistosoma* eggs and showed that vascular endothelial growth factor (VEGF) contributes to the TH2 environment that supports airway remodeling and vascular inflammation. SEA has also been utilized as an adjuvant to boost a cytotoxic lymphocyte population following vaccination with an HIV-1 Gag construct [14].

Last, and not least, we introduce *Schistosoma haematobium* to dissect the link between inflammation and cancer. The International Agency for Research in Cancer (IARC) of WHO has declared *S*. *haematobium* to be a class I carcinogen for the bladder, "definitely carcinogenic for humans" [15]. Despite the strong link between *S*. *haematobium*, the causative agent of urogenital schistosomiasis, and bladder carcinogenesis, we understand very little regarding how this helminth causes cancer. Most hypotheses suggest that *S*. *haematobium-*induced chronic inflammation is critical in the oncogenic process. Interestingly, *S*. *haematobium* is linked to higher rates of squamous cell carcinoma of the bladder, a form of bladder cancer that is unusual in non-*S*. *haematobium-*infected patients. Our inadequate knowledge of schistosomal bladder carcinogenesis underscores the importance of tractable animal models of urogenital schistosomiasis [16–23]. These models have identified potential mediators of fibrosis, immunomodulation, and carcinogenesis that may be common to multiple neoplasms. Given the evidence that urogenital schistosomiasis-induced bladder cancer is an inflammatory process, studying schistosomiasis, even if eradicated, may reveal new links between inflammation and carcinogenesis that are suitable as therapeutic targets in non-schistosomal cancers. Thus, prudent laboratory maintenance and study of particular pathogens that have been globally eliminated or eradicated may yield unique scientific discoveries of broad significance.
